# Trends of nucleic acid – based point-of-care diagnostics for infectious diseases

**DOI:** 10.1186/s13036-026-00681-6

**Published:** 2026-04-29

**Authors:** Hossam Hatem, Mohamed Mysara, Raghda Ramadan

**Affiliations:** 1 Bioinformatics Group, Center for Informatics Science (CIS), School of Information Technology and Computer Science (ITCS), Nile University, Giza, 12588 Egypt; 2 Biotechnology Research and Innovation Centre (BRIC), School of Biotechnology, Nile University, Giza, 12588 Egypt

**Keywords:** Point-of-care diagnostics, Nucleic Acid-based Diagnostics, Infectious Disease, lab-on-a-chip, CRISPR-based Diagnostics

## Abstract

The global emergence and spread of infectious diseases highlights the importance of having an easily accessible, decentralized testing modality that is adept at quickly identifying the nucleic acids present in a Point-of-Care (PoC) setting. PoC-based nucleic acid diagnostics encompass a broad range of platforms, such as lateral flow, biochips, and biosensors, whose primary goal has always been to offer sensitive, selective, and economically viable testing beyond conventional laboratory facilities. The current review illustrates a comprehensive overview of PoC-based nucleic acid diagnostics for infectious diseases, as it is divided into three major operational steps: nucleic acid extraction, amplification, and detection. Firstly, it describes strategies that could effectively work as a PoC, such as magnetic bead-based, paper-based, and integrated microfluidic approaches, while further focusing on their ability to remain simplified, rugged, and equipment-free. Secondly, it summarizes key amplification methods, such as Polymerase Chain Reaction (PCR), Nucleic Acid Sequence-Based Amplification (NASBA), Recombinase Polymerase Amplification (RPA), and Loop-Mediated Isothermal Amplification (LAMP), as they have all been modified to effectively work under more rapid, low-power, and portable conditions. Lastly, it introduces all presently known detection platforms, starting from simpler colorimetric and fluorescent assays present in lateral flow or device platforms, to innovative and advanced biosensors like those exploiting CRISPR/Cas systems and toehold switch principles, as these detectively offer highly sensitive and highly programmable nucleic acid recognition.

**Graphical Abstract:**

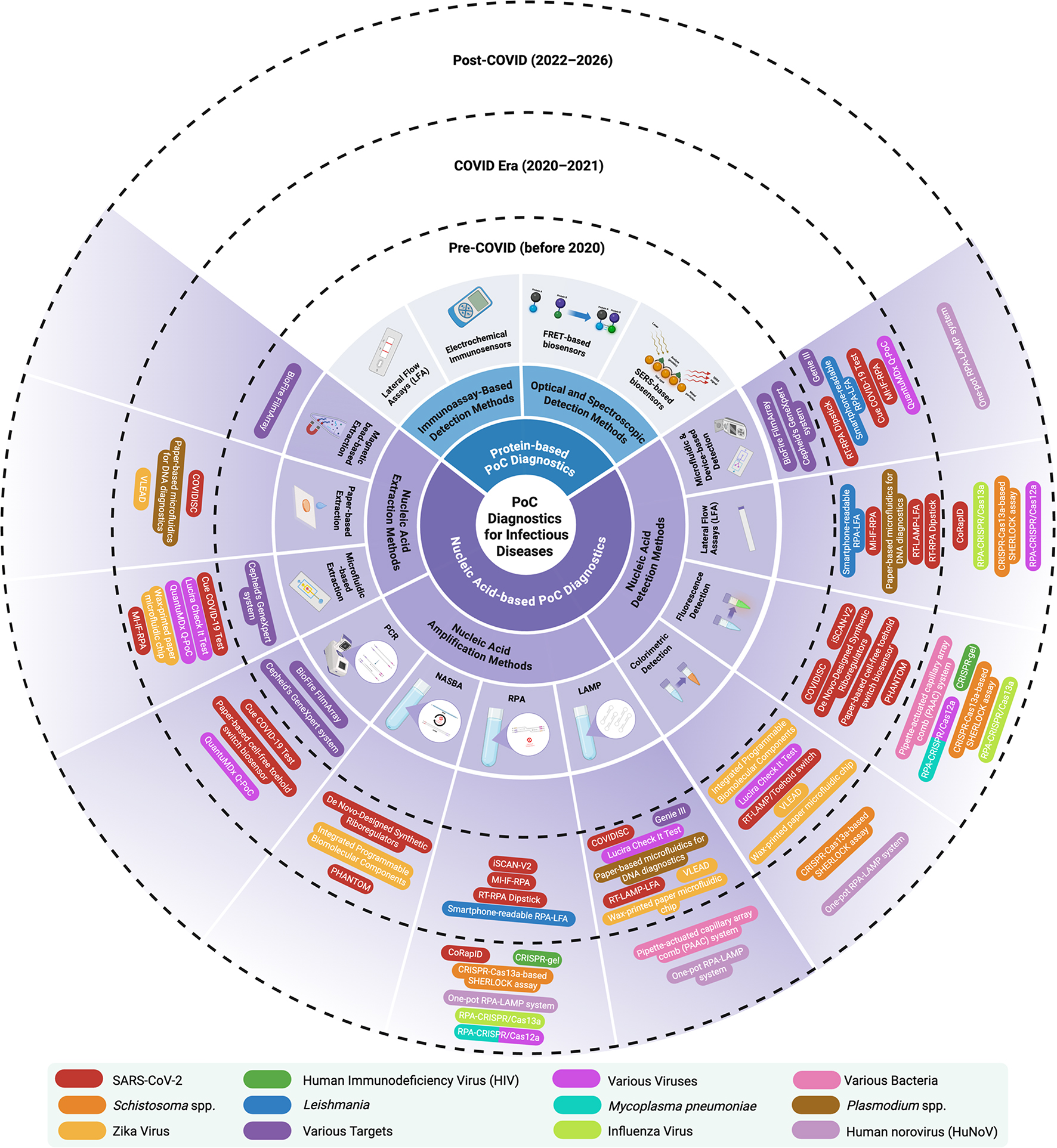

## Introduction

Over the past two decades, several pandemics have emerged, including Severe Acute Respiratory Syndrome (SARS), Ebola virus, Middle East Respiratory Syndrome (MERS), Zika virus, and, most recently, Severe Acute Respiratory Syndrome Coronavirus 2 (SARS-CoV-2). These epidemics have highlighted weaknesses in global healthcare systems and recorded an urgent demand for medical innovations. The recurring nature of these crises indicates the critical demand for rapid, accurate, cost-effective, and accessible diagnostic tools, particularly in resource-limited settings [1, 2]. As cited by the World Health Organization (WHO), the global spread of infectious diseases is steadily increasing due to several reasons, including microbial resistance and zoonotic spillover [3]. Delays in identifying and controlling infectious diseases exacerbate public health challenges, underscoring the need for innovative point-of-care (PoC) diagnostics to enhance outbreak preparedness and response [4].

Accurate and accessible diagnostic tools are crucial for the successful management of infectious diseases that pose a great threat globally. However, traditional diagnostics face significant drawbacks, including delayed results, high expenses, and dependence on advanced infrastructure and skilled staff. These are not suitable for resource-limited settings or urgent cases. Therefore, there is a pressing need for diagnostic solutions that are rapid, reliable, and accessible to patients regardless of their location [5]. To address these drawbacks, PoC testing has been proven to be a new approach towards ensuring rapid, near-patient detection of pathogens with minimal infrastructure. Recent advancements in PoC technology have dramatically improved the sensitivity, specificity, and cost-efficiency of these tools, making them increasingly vital in managing emerging and re-emerging infectious diseases [6, 7].

**Fig. 1:**
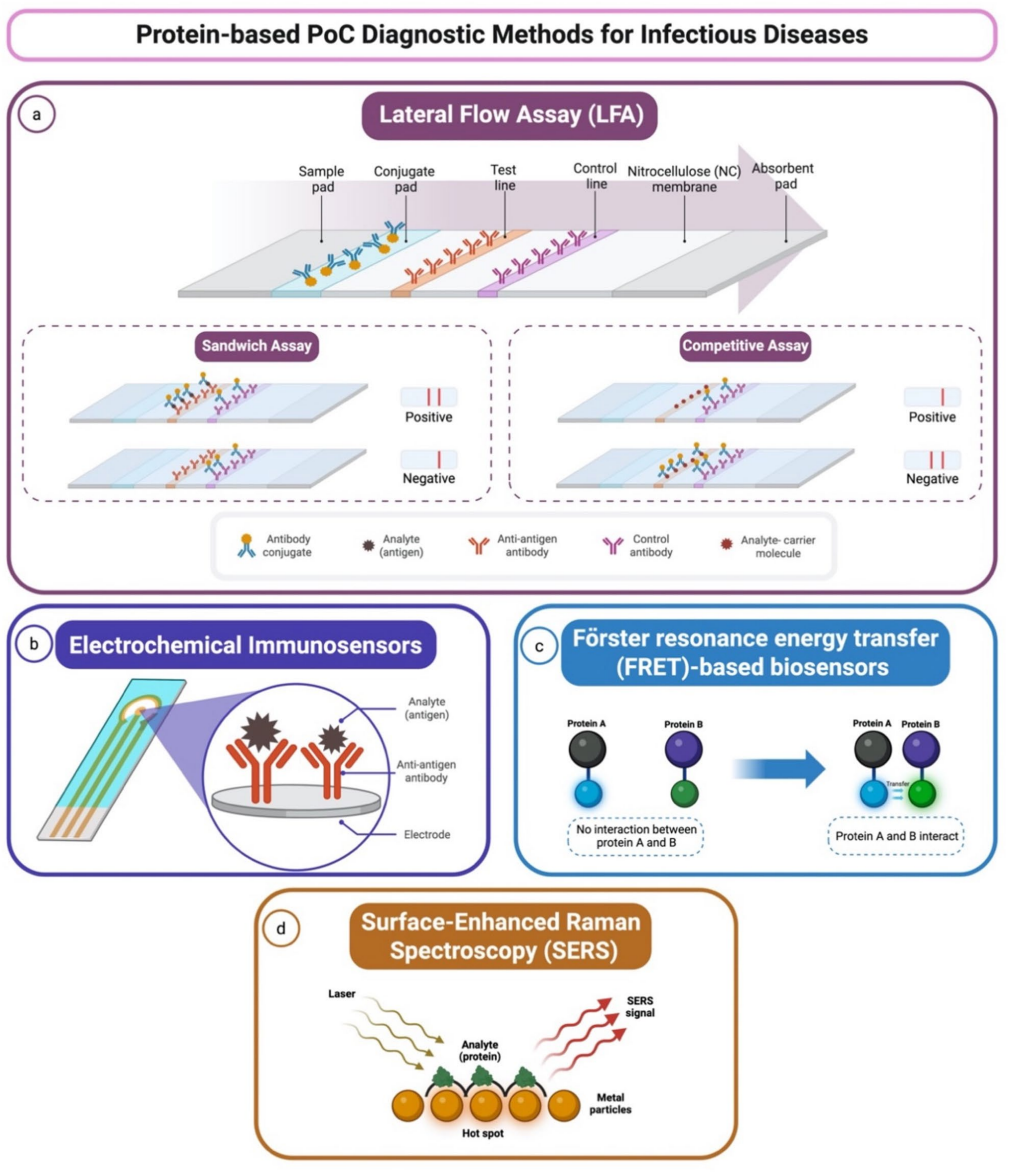
Protein-based point-of-care (PoC) diagnostic methods for infectious diseases. This figure illustrates various PoC diagnostic methods designed for detecting infectious diseases. (**a**) Lateral Flow Assay (LFA): The diagram shows the structure of an LFA strip, comprising a sample pad, conjugate pad, test line, control line, nitrocellulose (NC) membrane, and absorbent pad. Two assay formats are presented: the sandwich assay, where analyte binding results in a positive signal, and the competitive assay, where the absence of a signal indicates a positive result. (**b**) Electrochemical Immunosensors: A sensor designed to detect biomolecules through antibody-antigen interactions, producing measurable electrical signals for diagnostic applications. (**c**) Förster Resonance Energy Transfer (FRET)-based Biosensors: the detection mechanism depends on energy transfer between two fluorophores (Protein A and Protein B), indicating molecular interaction. The signaling indicates a target analyte. (**d**) Surface-Enhanced Raman Spectroscopy (SERS): Illustration of SERS technology, where analytes are detected via enhanced Raman signals generated by metal nanoparticles acting as signal amplifiers at electromagnetic “hot spots”. Created with Biorender.com

PoC diagnostic tests can be broadly categorized based on their detection mechanisms, with protein-based and nucleic acid-based approaches being the two primary types. Protein-based diagnostics function by detecting pathogen-specific antigens or host antibodies, making them valuable for identifying active infections and immune responses [8]. Among these, lateral flow assays (LFAs) represent the most widely adopted format. LFAs are widely used for a range of infections, including HIV, influenza, dengue, and most recently, SARS-CoV-2, owing to their low cost, portability, and user-friendliness (Fig. 1a). LFAs typically operate in either a sandwich format for antigen detection or a competitive format for small molecule or antibody detection, offering qualitative or semi-quantitative results [9]. Beyond LFAs, protein-based PoC diagnostics have evolved to include a variety of biosensing platforms. Electrochemical immunosensors convert antigen-antibody interactions into electrical signals, enabling quantitative and multiplexed detection in compact, low-power formats suitable for integration with mobile health technologies (Fig. 1b) [10]. Förster Resonance Energy Transfer (FRET)-based biosensors enable real-time detection of molecular interactions through changes in fluorescence energy transfer, allowing for precise identification of target analytes (Fig. 1c) [11–15]. Similarly, Surface-Enhanced Raman Scattering (SERS) platforms leverage signal amplification by metallic nanostructures to detect low-abundance proteins with high specificity and spectral resolution (Fig. 1d) [16–20].

Despite these advancements, protein-based diagnostics are frequently challenged by issues of low sensitivity, particularly in early infections when antigen or antibody levels are significantly low. Antigen levels may fluctuate significantly across the course of an infection, and antibody responses often lag behind symptom onset, making early diagnosis unreliable in many cases. Additionally, cross-reactivity among structurally similar antigens or non-specific binding can reduce diagnostic specificity, especially in regions endemic to multiple co-circulating pathogens [21]. This drawback reduces their effectiveness in early disease detection, highlighting the need for complementary diagnostic systems that can improve overall diagnostic accuracy.

Nucleic acid diagnostics, on the other hand, almost exclusively target the nucleic acids of pathogens (DNA or RNA) and are therefore highly reliable for diagnosing and monitoring infectious diseases [22]. However, this targeted approach presents limitations when the causative pathogen is unknown. In such cases, broad-range or metagenomic approaches, such as shotgun next-generation sequencing (NGS) can be employed to detect and identify novel or unanticipated pathogens by analyzing the entire nucleic acid content of a sample without prior knowledge of the target sequence [23]. These untargeted strategies are typically implemented in centralized or minimized laboratory settings like portable sequencing or metagenomic workflows [24, 25]. While these untargeted strategies sacrifice some of the speed and simplicity advantages of pathogen-specific PoC assays, they represent an important complementary tool in outbreak scenarios [26]. Established amplification methods, such as the Polymerase Chain Reaction (PCR), remain the gold standard for diseases like COVID-19, tuberculosis, and HIV due to their diagnostic accuracy [27, 28]. Besides, new amplification technologies such as Loop-mediated Isothermal Amplification (LAMP), Recombinase Polymerase Amplification (RPA), and Nucleic Acid Sequence-based Amplification (NASBA) are changing PoC testing by enabling rapid and low-cost identification [29–31]. Furthermore, Clustered Regularly Interspaced Short Palindromic Repeats (CRISPR)-based diagnostic platforms have emerged as highly sensitive, programmable tools for nucleic acid detection, further enhancing PoC capabilities [32, 33]. Despite their high sensitivity and specificity, nucleic acid-based diagnostics often require complex instrumentation and costly reagents, limiting their practicality in low-resource and emergency settings [34–37].

In this review, we focus on the landscape of nucleic acid-based PoC diagnostic methods, highlighting their principles, applications, and limitations, providing a comprehensive and integrated perspective on the full PoC nucleic acid diagnostic pipeline. Building on this, we first outline the current diagnostic landscape and core methodological pillars (extraction, amplification, and detection), and then introduce the operating principles and mechanisms of innovative PoC technologies such as CRISPR/Cas and toehold switch systems, highlighting their advantages and their potential integration into modern diagnostic approaches and digital health frameworks.

## Part I: The diagnostic landscape and conceptual framework

### Overview of nucleic acid-based PoC diagnostics

**Fig. 2:**
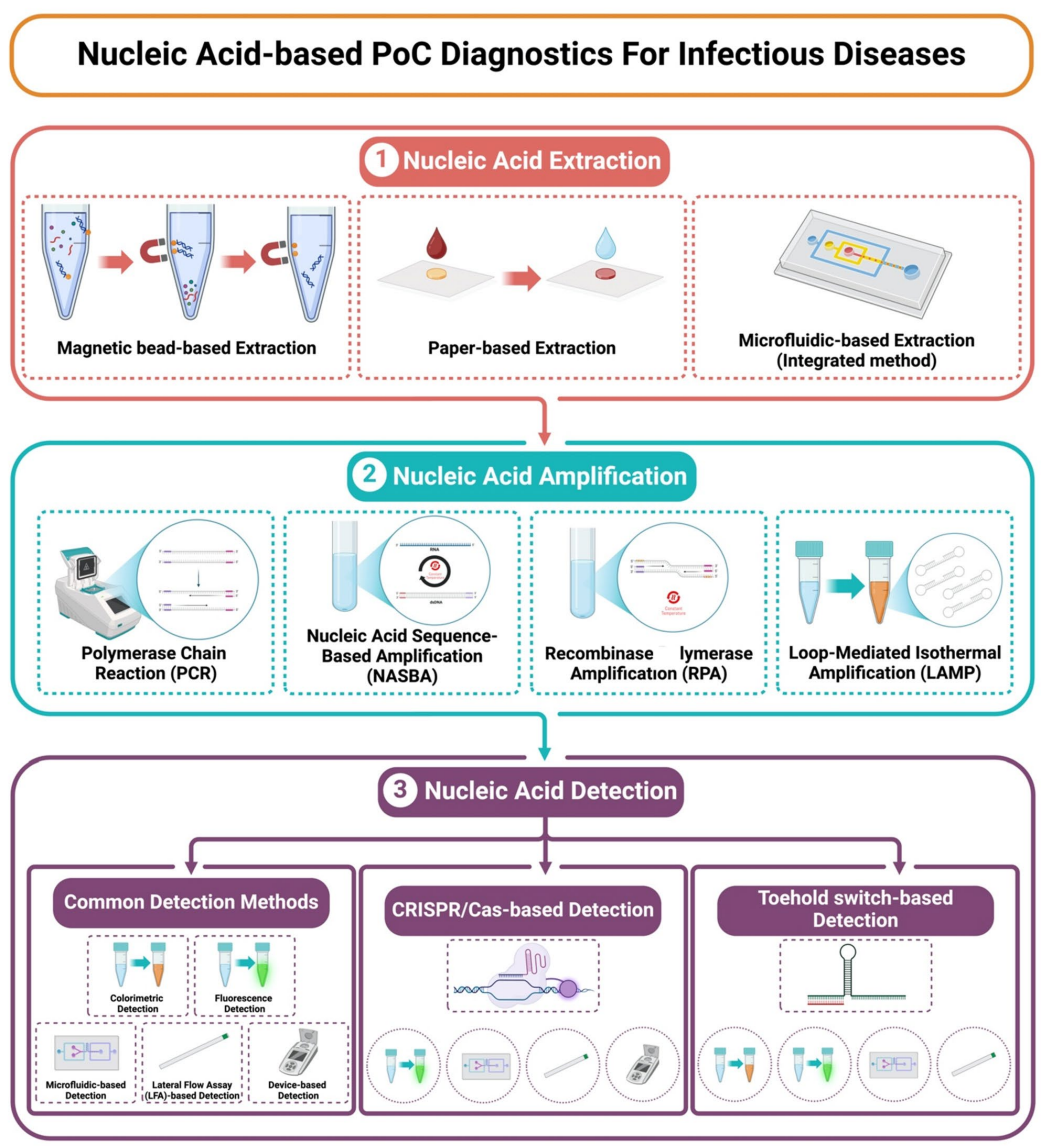
Graphical illustration summarizing nucleic acid-based point-of-care (PoC) diagnostics for infectious diseases. Created with Biorender.com

Nucleic acid-based PoC diagnostics facilitate a rapid, sensitive, and accurate detection of infectious diseases, allowing early clinical decision-making. These systems typically involve three essential steps: nucleic acid extraction, amplification, and detection, as described in (Fig. 2). The extraction step isolates DNA or RNA from various clinical specimens, such as blood, saliva, oropharyngeal swabs, or urine, purifying away potential inhibitors that could compromise downstream processes. Rapid and efficient methodologies such as magnetic bead-based and paper-based extraction are prevalent for this process [38, 39]. Following extraction, target nucleic acids are amplified by methods such as polymerase chain reaction (PCR), Nucleic Acid Sequence-Based Amplification (NASBA), recombinase polymerase amplification (RPA), or loop-mediated isothermal amplification (LAMP). These methods increase the copy number of the target sequence to easily interpretable quantities. Finally, amplified nucleic acids are detected based on various detection modalities, such as colorimetric, fluorescent assays, electrochemical or lateral flow assays (LFA), generating results in formats conducive to PoC utilization. Collectively, these integrated approaches yield diagnostic technologies that are accurate, rapid, and cost-effective, making them ideal for decentralized healthcare systems [37, 40, 41]. In recent years, particularly during and after the COVID‑19 pandemic, there has been a marked increase in research and publication activity focused on innovative nucleic acid-based PoC platforms, reflecting growing interest in scalable, outbreak-responsive diagnostic solutions. (Table 1) provides a comparative snapshot of nucleic acid-based diagnostic technologies for infectious diseases, including amplification modes, detection formats, target pathogens, and PoC compatibility levels, ranging from fully integrated commercial systems to promising, in-development versions.

**Table 1:** Summary of nucleic acid-based diagnostic platforms with point-of-care (PoC) applicability for infectious diseases

Method	Amplification/ Detection Technology	Target Pathogen	Sample Type	LOD/Sensitivity	Time (minute)	PoC Compatibility	Regulatory / Commercialization Status	References
BioFire FilmArray	Multiplex PCR/ Device-based	More than 30 targets, including RSV, SARS-CoV-2, and influenza	Sputum Endotracheal aspirate, Bronchoalveolar lavage Blood	varies by target	< 120	Near-PoC	FDA-Approved, Commercially available	[42]
Cepheid’s GeneXpert system	PCR/ Device-based	Various targets, including SARS-CoV-2 , RSV, and the influenza virus	Nasopharyngeal swab Sputum Blood Urine	varies by target	< 60	Near-PoC	CLIA-Waived, Commercially available	[43]
CoRapID	RT-RPA/ LFA	SARS-CoV-2 (Alpha to omicron variants)	Nasopharyngeal swab Oropharyngeal swab	1 copy/reaction (88.1% sensitivity)	30	True PoC	Commercially available	[44]
COVIDISC	RT-LAMP integrated with Intercalating dyes (SYTO-82) or fluorescent probes	SARS-CoV-2	Nasopharyngeal swab	1 copy/µL	20–60	True PoC	Commercially available	[45]
CRISPR-Cas13a-based SHERLOCK assay	RPA integrated with CRISPR/Cas13a Fluorescence and colorimetric (lateral flow)	*S. japonicum* *S. mansoni*	Faecal and serum samples from humans and mice	0.1 pg/µL for S. mansoni; 0.2 pg/µL for S. japonicum (100% sensitivity)	-	PoC-Compatible Prototype	-	[32]
CRISPR-gel	RT-RPA integrated with CRISPR-Cas12a	HIV	Plasma	30 copies per reaction	30	Prototype	-	[46]
Cue COVID-19 Test	NAAT (not specified)/ Electrochemical signal detection and connected to the Cue Health App	SARS-CoV-2	Nasopharyngeal swab	20 copies per reaction (100% sensitivity)	25	True PoC	Authorized for emergency use by the FDA and Health Canada Commercially available	[47]
De Novo-Designed Synthetic Riboregulators	NASBA integrated with the Toehold switch-based sensor with fluorescence detection	SARS-CoV-2	Nasopharyngeal swab	-	-	Prototype	-	[48]
Genie^®^ III	LAMP/ Device-based detection	*Chlamydia psittaci*,* Chlamydia pecorum*, SARS-CoV-2, and various targets	Nasopharyngeal swab Serum	-	30–60	Near-PoC	Commercially available	[49, 50]
Integrated Programmable Biomolecular Components	NASBA/ Integrated Toehold switch and CRISPR/Cas9 sensors with colorimetric detection	Zika virus and its African and American strains	Plasma	2.8 fM	-	Prototype	-	[51]
iSCAN-V2: one-pot RPA-CRISBR/EsCas13d	RT-RPA integrated with fluorescent signal using CRISBR/EsCas13d	SARS-CoV-2	Nasopharyngeal swab Oropharyngeal swab	8 copies/µl (200 copies/reaction) (93.75% sensitivity)	< 60	PoC-Compatible Prototype	-	[52]
Lucira Check It Test	RT-LAMP integrated with Colorimetric detection	SARS-CoV-2 Influenza virus A/B	Nasopharyngeal swab	93%	30	True PoC	Commercially available	[53]
Microfluidic-integrated RPA (MI-IF-RPA)	RT-RPA integrated with Microfluidic LF	SARS-CoV-2	Nasopharyngeal swab	30 copy/reaction (97% sensitivity)	30	PoC-Compatible Prototype	-	[54]
One-pot RPA-LAMP system	RPA/LAMP integrated with colorimetric microfluidic -based detection	HuNoV	Not specified	10 copies/µL (GI), 1 copy/µL (GII)	-	PoC-Compatible Prototype	-	[55]
paper-based cell-free toehold switch biosensor	Q5 DNA Polymerase (NEB) integrated with Toehold switch-based sensor with Bioluminescence detection.	SARS-CoV-2	Saliva	60 nM	7	PoC-Compatible Prototype	-	[56]
Paper-based microfluidics for DNA diagnostics	LAMP integrated with LFA	*P. vivax* *P.malariae* *P. ovale*	Blood	10⁵ IU/mL (98% sensitivity for *Plasmodium pan* and 93% sensitivity for *P. falciparum)*	< 50	PoC-Compatible Prototype	-	[57]
PHAsed NASBA-Translation Optical Method (PHANTOM)	NASBA integrated with the Toehold switch-based sensor with luminescence detection	SARS-CoV-2	Nasopharyngeal swab	100 copies/ reaction (85% sensitivity)	35	Prototype	Indian Complete Patent Application No. 202,041,030,231	[58]
pipette-actuated capillary array comb (PAAC) system	LAMP/ fluorescence-based detection	*E. coli* *K. pneumoniae* *S. aureus*	Urine	200, 500, and 500 copies, respectively	85	True PoC	-	[59]
QuantuMDx Q-PoC	RT-PCR/ device-based detection	SARS-CoV-2 Flu A/B RSV	Nasopharyngeal swab	1 copy/µL (96.88% sensitivity)	30	Near-PoC	Commercially available	[60]
RPA -CRISPR/Cas13a	RPA integrated with CRISPR/Cas13a Fluorescence readout	AIV specifically H1–H16 subtypes	Swab (samples from chickens)	69 copies/µL	-	PoC-Compatible Prototype	-	[61]
RPA -CRISPR/Cas13a	RPA integrated with CRISPR/Cas13a and LFA	AIV specifically H1–H16 subtypes	Oropharyngeal swab Nasopharyngeal swab	690 copies/µL	-	PoC-Compatible Prototype	-	[61]
RPA-CRISPR/Cas12a-based method	RPA integrated with CRISPR/Cas12a (fluorescent color visualized by the naked eye)	*M. pneumoniae*	Oropharyngeal swab, Bronchoalveolar lavage fluid Sputum	2 copy/reaction (99.1% sensitivity)	< 60	PoC-Compatible Prototype	-	[62]
RPA-CRISPR/Cas12a-based method	RPA integrated with CRISPR/Cas12a Fluorescence readout	HuNoV, HmPV, HBoV, SEoV, and RSV	Clinical samples (not specified)	9.65 × 10^2^ copies/mL	40	PoC-Compatible Prototype	-	[63]
RPA-CRISPR/Cas12a-based method	RPA integrated with CRISPR/Cas12a and LFA	HuNoV, HmPV, HBoV, SEoV, and RSV.	Clinical samples (not specified)	9.65 × 10^2^ copies/mL	40	PoC-Compatible Prototype	-	[63]
RT-LAMP-LFA	LAMP integrated with LFA	SARS-CoV-2	Oropharyngeal swab Nasopharyngeal swab	4 to 4 0 × 10-6 ng/µl (100% sensitivity)	75–90	PoC-Compatible Prototype	-	[64]
RT-LAMP/Toehold switch-based detection	RT-LAMP integrated with Toehold switch-based sensor with colorimetric detection	MERS-CoV, SARS-CoV-2	Saliva Respiratory tract samples	120 copies/ reaction	70	Prototype	-	[65]
-RT-RPA Dipstick using a smartphone	RT-RPA integrated with the Dipstick-based smartphone method	SARS-CoV SARS-CoV-2 MERS-CoV	Saliva	95% sensitivity	30	PoC-Compatible Prototype	-	[66]
Smartphone-readable RPA-LFA	RPA integrated with LFA	*Leishmania*	Blood	0.01 parasites per reaction (98.7% sensitivity)	-	PoC-Compatible Prototype	-	[30]
valve-enabled lysis, paper-based RNA enrichment, and RNA amplification device (VLEAD)	RT-LAMP and detected with the naked eye or a cellphone camera	Zika virus	Urine Saliva	0.5 PFU	50	PoC-Compatible Prototype	-	[67]
wax-printed paper microfluidic chip	RT-LAMP/ colorimetric detection	Zika virus	Urine Plasma	1 copy/µL	15	PoC-Compatible Prototype	-	[68]

RSV, Respiratory syncytial virus; SARS-CoV-2, Severe acute respiratory syndrome coronavirus 2; LAMP, Loop-mediated isothermal amplification; RPA, Recombinase polymerase amplification; LFA, Lateral flow assay; NAAT, Nucleic acid amplification test; NASBA, Nucleic acid sequence-based amplification; PoC, Point-of-care; MERS-CoV, Middle East respiratory syndrome coronavirus; HuNoV, Human norovirus; HBoV, Human bocavirus; HmPV, Human metapneumovirus; SEoV, Seoul virus; AIV, Avian influenza virus; *S. japonicum*,* Schistosoma japonicum; S. mansoni*,* Schistosoma mansoni; P. vivax*,* Plasmodium vivax; P. malariae*,* Plasmodium malariae; P. ovale*,* Plasmodium ovale; E. coli*,* Escherichia coli; K. pneumoniae*,* Klebsiella pneumoniae; M. pneumoniae*,* Mycoplasma pneumoniae ; S. aureus*,* Staphylococcus aureus;* VLEAD, Valve-enabled lysis, enrichment and amplification device; SYTO-82, Intercalating dye used in fluorescence detection. PoC Compatibility Categories: True PoC, fully portable and field-usable with minimal equipment; Near-PoC, requires device/infrastructure but usable in decentralized settings; PoC-Compatible Prototype, promising for PoC use but still under development; Prototype, early-stage and not yet PoC-ready

## Part II: Core methodological pillars: From sample to result

### Nucleic acid extraction strategies for PoC diagnostics

Nucleic acid extraction is a crucial step in molecular diagnostics, significantly impacting the sensitivity and specificity of downstream amplification and detection assays. Effective extraction methods isolate DNA or RNA from diverse biological samples while removing inhibitors that could compromise enzymatic reactions and assay performance. In resource-limited settings, conventional lab-based methods—often reliant on centrifugation steps, toxic chemicals, complex multi-step protocols, and well-trained personnel—are unsuitable for PoC diagnostics [39]. For PoC applications, extraction methods must be simple, affordable, and robust, maintaining efficiency under operational and environmental constraints. Key challenges faced during the development and implementation of nucleic acid extraction methods in resource-limited settings include the lack of electricity or storage units like refrigerators, using large and advanced equipment like centrifuges and automated extractors, high expenses, environmental factors such as extreme temperatures, humidity and dust which affect the efficiency of the used reagent, and lack of trained staff. To address these challenges, the ideal PoC nucleic acid extraction methods should minimize protocol steps, reduce or eliminate the dependence on electricity, and be stable as long as possible at room temperature [69].

Current PoC extraction methods are typically divided into three types: magnetic bead–, paper–based, and integrated methods (microfluidic-based). Magnetic bead–based extraction utilizes functionalized magnetic particles to bind nucleic acids from complex clinical samples selectively, enabling rapid isolation and minimal instrumentation [39]. Paper-based extraction utilizes porous materials, such as filter paper or modified membranes, to capture and purify nucleic acids at a low cost and without the need for complex equipment [70]. In contrast, the integrated method often utilizes advanced microfluidic systems that consolidate sample preparation, extraction, amplification, and detection within a single, cohesive platform, enabling truly decentralized diagnostics [59]. As summarized in (Table 2), recent advances in PoC nucleic acid extraction methods are highlighted, detailing the extraction technology employed, sensitivity, applicable sample types, target pathogens, and PoC compatibility.

**Table 2:** Recent advanced methods for nucleic acid extraction and their point-of-care (PoC) compatibility status

Extraction method	Method	Sample type	Sensitivity	Time (minute)	Target pathogen	PoC Compatibility	References
Magnetic bead-based	SPE using Magnetic beads	Blood	10^2^ copies/mL	15	HBV	True PoC	[71]
	SPE using a magnetic bead	Saliva	91.3%	10	*M.tb*	Near-PoC	[72]
	SPE using a magnetic bead	Saliva	-	< 15	*C. trachomatis*, Zika, and Dengue Virus	Near-PoC	[73]
	Magnetic bead-based DNA extraction into a transfer pipette	Sputum Urine	10% for sputum and 90% for urine	20	*M. tb*	PoC-Compatible Prototype	[39]
	Easy Express Extraction (Triple*E*)	Blood Serum oral/nasal swabs	Viral detection up to a 10^− 4^ dilution	6–10	African swine fever, lumpy skin disease, Bluetongue, and Morbillivirus	PoC-Compatible Prototype	[69]
Paper-based	Glass filter membrane (GF/F grade, Whatman) captured DNA	Blood Saliva Urine	-	3	*S. aureus*	True PoC	[70]
	Sequence-specific capture of nucleic acids on a glass fiber membrane	Serum	The method shows 10 times less sensitivity in human serum compared to PBS buffer	5	Zika, Dengue, and Chikungunya virus	True PoC	[74]
	Cellulose paper-based RNA extraction	Urine Saliva	achieves comparable sensitivity with a commercial SPE-based RNA extraction kit	-	Zika virus	True PoC	[67]
	Chitosan-modified silicon dioxide capillaries	Saliva	50 TU/mL	25	Zika virus	Near-PoC	[75]
	Paper origami device	Blood	-	5	Malaria	True PoC	[57]
	Cellulose filter papers with surfactant (DBSFP)	Blood	-	< 7	*P. falciparum*	PoC-Compatible Prototype	[38]
	Chitosan-modified Fusion 5 membrane captured DNA	Urine	-	2	*TV*	Prototype	[76]
Integrated method (microfluidic-based)	Chitosan-modified glass filter paper embedded in capillaries captured nucleic acids.	Urine	97%	-	*E. coli*,* K. pneumoniae*, and *S. aureus*	PoC-Compatible Prototype	[59]
	Ultrasonic-assisted magnetic beads-based SPE	Serum	10^3^ copies HBV/mL 5 × 10^3^ copies HIV/mL	< 1	HBV HIV	PoC-Compatible Prototype	[77]
	Wax wax-printed paper microfluidic chip was used to filter target pathogens.	Urine Plasma	-	-	Zika virus	PoC-Compatible Prototype	[68]
	Surface-modified microfluidic chip	Serum	90%	30	*E. coli*	PoC-Compatible Prototype	[78]
	SPE using magnetic particles	Oropharyngeal swabs	consistent with the conventional method	15	*S. pneumoniae and M. pneumoniae*	PoC-Compatible Prototype	[79]
	Chitosan-modified Fusion 5 filter paper captured viral RNA	Plasma	-	10	Dengue virus	Near-PoC	[80]

HBV, Hepatitis B virus; HIV, Human immunodeficiency virus; C. trachomatis, Chlamydia trachomatis; K. pneumoniae, Klebsiella pneumoniae; M. tb, Mycobacterium tuberculosis; S. pneumoniae, Streptococcus pneumoniae; M. pneumoniae, Mycoplasma pneumoniae; S. aureus, Staphylococcus aureus; E. coli, Escherichia coli; TV, Trichomonas vaginalis; DBSFP, dried blood spot filter paper; SPE, solid-phase extraction; TU, transducing units. PoC Compatibility Categories: True PoC, fully portable and field-usable with minimal equipment; Near-PoC, requires device/infrastructure but usable in decentralized settings; PoC-Compatible Prototype, promising for PoC use but still under development; Prototype, early-stage and not yet PoC-ready

#### Magnetic Bead-based Method

Magnetic bead-based extraction methods have emerged as a practical and versatile approach for PoC diagnostics due to their high efficiency, scalability, and adaptability to miniaturized systems [81, 82]. These techniques rely on functionalized magnetic beads, typically coated with silica or carboxyl groups, which bind to nucleic acids under specific buffer conditions, such as high concentrations of chaotropic salts [83]. Upon binding, the beads are magnetically separated from the sample matrix, allowing for rapid washing and elution without the need for centrifugation or filtration. This simplicity facilitates integration into portable diagnostic systems and supports a broad range of clinical samples, including sputum, saliva, blood, urine, and swabs [84]. Several studies highlight the method’s versatility. For instance, Chan et al. (2018) reported a solid phase extraction (SPE) approach that achieved high nucleic acid recovery from saliva in under 15 min [73]. Similarly, Pearlman et al. (2020) reported efficient recovery of *Mycobacterium tuberculosis (M. tuberculosis*) DNA from sputum and urine, achieving sensitivities of 10% and 90%, respectively [39]. Another new method, known as Easy Express Extraction (TripleE), has also been developed, enabling the high-speed extraction of nucleic acids (6–10 min) from clinical specimens, including serum, swabs, and blood. The method has had practical application in extracting viral nucleic acids, holding much potential for PoC diagnostics in resource-limited settings [69].

Magnetic bead extraction has found widespread application on various target pathogens in PoC diagnostics. For SARS-CoV-2, magnetic bead-based extraction of nasopharyngeal swabs, in conjunction with RT-qPCR and RT-LAMP assays, has been shown to identify as few as 10–100 viral copies/reaction with an overall assay time of 90 min [85]. In HIV diagnostics, magnetic bead protocols have been optimized for plasma RNA extraction, supporting isothermal amplification and decentralized viral load monitoring [86]. For flaviviruses such as dengue and Zika, magnetic bead-based RNA purification has enabled effective PoC detection using RPA and LAMP in settings lacking conventional laboratory infrastructure [73]. Furthermore, bead-based extraction of hepatitis B virus (HBV) DNA from blood samples has shown promise in integration with microfluidic systems for streamlined diagnosis in endemic regions [71]. These examples highlight the versatility and widespread applicability of magnetic bead-based extraction methods, which offer sensitive, rapid, and cost-effective diagnostic solutions for a vast majority of infectious pathogens. A significant advantage of magnetic bead systems is their compatibility with downstream amplification techniques, including PCR, LAMP, and RPA. Eluates generated using these protocols are typically free from enzymatic inhibitors, allowing seamless amplification. One illustrative study, presented by Bordelon et al. (2013), demonstrated that a silica-coated magnetic bead method recovered approximately 50 ± 5% of spiked M. tuberculosis IS6110 DNA from 1 mL urine while concentrating the DNA about tenfold (from 5 × 10⁵ to ~ 5 × 10⁶ copies/µL) and achieved an estimated LOD of 77 copies/µL. It showed performance comparable to commercial spin-column kits while using a simpler, equipment-free workflow [87].

Recent advancements are focusing on enhancing binding efficiency and minimizing inhibitor carryover through novel bead coatings, such as graphene oxide and zwitterionic polymers, thereby increasing the robustness of the assay [88, 89]. Moreover, research is exploring the use of functionalized magnetic nanoparticles conjugated with pathogen-specific aptamers or antibodies, which enables simultaneous pathogen capture and nucleic acid extraction, thereby streamlining the diagnostic workflow [90]. Additionally, integration with CRISPR/Cas-based systems is being explored, where magnetic beads serve not only as extraction reagents but also as target-specific signal amplification platforms. Despite these advancements, scalability and cost remain challenges for widespread implementation in resource-limited settings. As these technologies advance, magnetic bead-based systems are set to transform PoC diagnostics by providing a high-quality nucleic acid purification, a quick, and easy-to-use method [90, 91].

#### Paper-based extraction

Paper-based nucleic acid extraction methods have emerged as a viable solution for PoC diagnostics, owing to their low cost, simplicity, and suitability for resource-limited settings. These methods utilize various types of porous membranes, including cellulose, glass fiber, nitrocellulose, FTA cards, or silica-coated membranes, to bind nucleic acids through electrostatic forces, hydrogen bonding, or chaotropic salt-mediated adsorption. These extraction systems employ principles of capillary action, lateral flow, or filtration to extract DNA or RNA from various clinical samples, such as blood, saliva, urine, and swabs, without the need for elaborate instrumentation [74, 92–96]. Several studies highlight their utility. Seok et al. (2019) demonstrated that glass filter membranes can extract *S. aureus* DNA from various clinical specimens. In contrast, modified cellulose matrices have demonstrated high sensitivity for detecting Zika virus RNA in urine and saliva, comparable to that of commercial spin columns [67, 70].

Various paper-based extraction technologies have since been developed for capturing nucleic acids, which promise towards PoC diagnostics. Sequence-specific nucleic acid capturing based on glass fiber membranes stands out for highly sensitive detection of Zika virus, Dengue virus, and Chikungunya virus from serum specimens, exhibiting better efficiency compared to spin-column-based approaches [74]. Additionally, chitosan-modified silicon dioxide capillaries have also been employed to extract nucleic acids from saliva, enabling near-PoC detection of the Zika virus at a sensitivity of 50 TU/mL [75]. Other innovations include paper origami devices for blood-based malaria detection [57], cellulose filter papers with surfactants for blood-based *detection of P. falciparum* [38], and chitosan-modified Fusion 5 membranes for capturing DNA from urine, designed to detect *Trichomonas vaginalis* [76]. These methods highlight the growing potential of paper-based extraction technologies in enabling accessible, cost-effective, and rapid diagnostic solutions in low-resource environments.

While paper-based extraction methods offer benefits, they also have drawbacks, such as inconsistencies in nucleic acid recovery and sensitivity to environmental pollutants. To overcome these limitations, researchers have optimized buffer compositions, drying protocols, and membrane chemistries to enhance performance. For example, Margo et al. (2017) developed a paper device integrated with a lyophilized reagent for extracting Ebola virus RNA, which is stable at room temperature for more than six months [97]. Future directions involve incorporating automated readouts through smartphone imaging or portable fluorometers to facilitate comprehensive PoC diagnostics [97].

#### Integrated microfluidic extraction platforms

Integrated nucleic acid extraction methods combine sample preparation, target isolation, amplification, and detection into a single cohesive platform, enabling the rapid, accurate, and cost-effective diagnosis of infectious diseases at the PoC. By consolidating these steps, such platforms minimize handling errors, reduce cross-contamination, and eliminate the need for complex laboratory infrastructure or highly trained personnel [98, 99]. These integrated methods typically utilize microfluidic systems that contain embedded reagents and functionalized surfaces, as well as magnetic beads, for the processing of nucleic acids. Following sample input, on-chip lysis releases nucleic acids, which are captured using immobilized probes or beads. Sequential washing removes contaminants, and the purified nucleic acids are eluted for downstream amplification (e.g., LAMP, RPA) or detection assays [59]. Recent innovations highlight the versatility and diagnostic potential of these platforms. For example, a chitosan-modified glass filter paper embedded in capillaries has successfully detected *E. coli*,* Klebsiella pneumoniae*, and *S. aureus* in urine samples with 97% efficiency via smartphone-based readout [59]. Similarly, wax-printed paper microfluidic chips have enabled the isolation of pathogens from urine and plasma for the detection of the Zika virus. These innovative methods highlight the growing integration of functionalized materials and microfluidics to enhance extraction efficiency, making them valuable for rapid diagnostics and PoC applications [68].

Furthermore, microfluidic-integrated extraction systems have been developed for various diagnostic applications, including the detection of HIV and HBV in serum. These systems integrate extraction and amplification on a single chip, with one method achieving detection at concentrations of 10^3^ copies of HBV/mL and < 10^3^ copies of HIV/mL [77]. Additionally, a surface-modified chip achieved 90% efficiency in identifying *E. coli* in serum [78], while another platform using chitosan-treated Fusion 5 membranes effectively isolated dengue viral RNA from plasma [80]. These systems offer near-PoC diagnostic capability, especially valuable in resource-limited environments. Nevertheless, obstacles persist in enhancing bead-surface chemistry and buffer compositions to accommodate various biological samples (e.g., thick sputum or feces) [98].

### Nucleic acid amplification techniques for PoC diagnostics

#### Polymerase chain reaction (PCR)

**Fig. 3:**
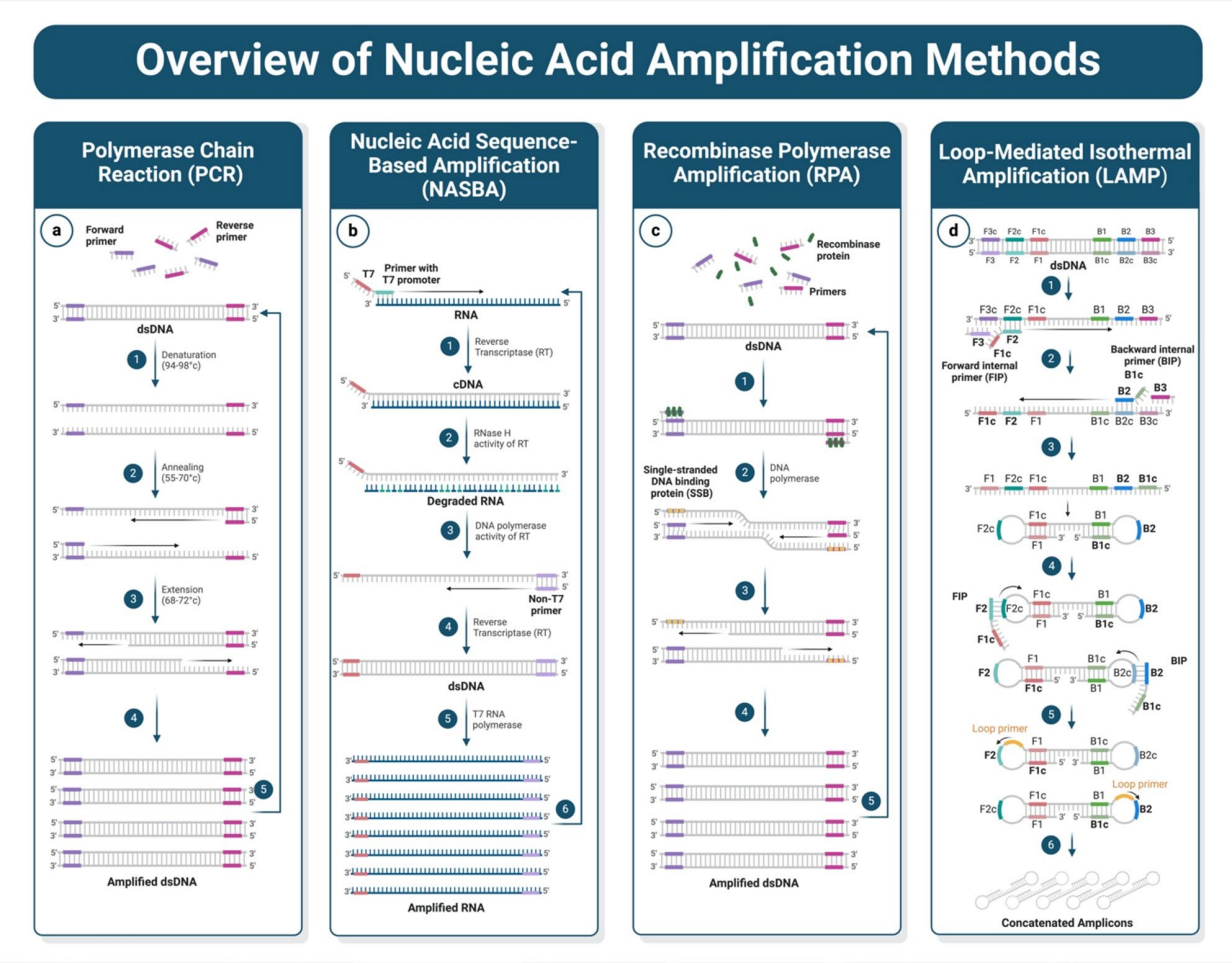
Overview of nucleic acid amplification methods**.** This figure illustrates the mechanisms of four of the most common nucleic acid amplification methods commonly used in point-of-care (PoC) diagnostics: (**a**) Polymerase Chain Reaction (PCR), this method amplifies double-stranded DNA (dsDNA) using two target sequence-discriminating primers (forward and reverse primers) and a DNA polymerase enzyme in three cycles: denaturation (94-98°C), annealing (55-70°C), and extension (68-72°C). (**b**) Nucleic Acid Sequence-Based Amplification (NASBA) is an isothermal method for amplifying RNA. It converts RNA to its complementary DNA (cDNA) using reverse transcriptase, which is later transcribed by RNA polymerase. (**c**) Recombinase Polymerase Amplification (RPA) is an isothermal amplification method that contains recombinase proteins, single-stranded DNA binding proteins (SSBs), and a strand-displacing DNA polymerase that amplifies dsDNA at low temperatures (typically 37–42°C). (**d**) Loop-Mediated Isothermal Amplification (LAMP) is a precise method of isothermal amplification. It employs several primers to form loop structures, which results in continuous DNA synthesis. It results in concatenated amplicons. Created with Biorender.com

Polymerase Chain Reaction (PCR) is considered the gold standard for nucleic acid amplification due to its exceptional sensitivity and specificity in detecting low viral loads, crucial for pathogens such as SARS-CoV-2, HIV, and HCV (Fig. 3a) [100–102]. Diagnostic adaptations, such as quantitative real-time PCR (qPCR), reverse transcription PCR (RT-PCR), and multiplex PCR, have enabled the simultaneous detection of multiple targets and the quantification of viral burden, thereby expanding the clinical utility of PCR [99, 100, 103]. Recent technologies have made possible adoption of PCR for PoC diagnostics possible by miniaturizing systems and simplifying workflows to reduce dependence on laboratory infrastructures and skilled personnel. For instance, BioFire FilmArray systems exemplify this shift, employing multiplex PCR in a self-contained cartridge format capable of identifying a broad spectrum of pathogens from respiratory, gastrointestinal, and cerebrospinal fluid samples, including RSV, SARS-CoV-2, influenza viruses, *S. pneumoniae*, and *M. pneumoniae* in under two hours. It provides a rapid and whole pathogen identification method that can serve as a near-PoC diagnostic tool [42, 104–107]. In the area of bloodstream infections, the BioFire Blood Culture Identification (BCID) panel identifies more than 24 pathogens and resistance markers from blood culture bottles immediately, making it valuable in sepsis management [106]. 

Similarly, Cepheid’s GeneXpert system integrates sample preparation and qPCR detection for various infectious agents across multiple specimen types, with results typically available within one hour [43, 108]. Despite their advantages, these systems are limited for PoC purposes in resource-limited conditions due to their bulkiness, cost, and the necessity for technical personnel. To address these constraints, next-generation platforms, such as the CLIA-waived QuantuMDx Q-PoC, have emerged. This compact system performs multiplex PCR directly from nasal or oropharyngeal swabs, detecting SARS-CoV-2, influenza A/B, and RSV within 30 min, with an LOD of 1 copy/µL and sensitivity approaching 97% [28, 60, 109]. Additionally, alternative heating technologies, including thin-film resistive heaters and convective PCR systems, have been explored to eliminate the need for bulky thermocyclers, thereby enhancing PCR’s feasibility for PoC settings [110]. These innovations enable faster and more compact thermal cycling, making PCR more practical for decentralized diagnostic applications. Nonetheless, isothermal alternatives like LAMP and RPA are inherently better suited for PoC use, as they operate at constant temperatures with minimal equipment.

#### Nucleic acid sequence-based amplification (NASBA)

NASBA is a widely used isothermal amplification method operating at ~ 41 °C, making it well-suited for point-of-care (PoC) diagnostics in low-resource settings. The NASBA amplification process begins with the binding of a T7 promoter to the target RNA, facilitating the synthesis of cDNA using the reverse transcriptase enzyme (RT) (Fig. 3b). The RNase H activity of RT degrades the RNA to build the dsDNA using the DNA polymerase activity of RT. A non-T7 primer is used by reverse transcriptase to generate a new cDNA strand. Ultimately, T7 RNA polymerase recognizes the T7 promoter and initiates RNA transcription, resulting in amplified RNA copies that can be detected using various detection and visualization methods [33, 110]. Its application spans a wide range of pathogens, including malaria, SARS-CoV-2, and Zika virus, and it is compatible with advanced detection tools such as CRISPR/Cas systems and toehold switch sensors [31, 51, 111]. Commercially, NASBA has been adopted in laboratory-based platforms, such as the NucliSens EasyQ system by bioMérieux, for HIV RNA quantification, demonstrating its clinical utility in viral load monitoring [112].

Although no fully integrated NASBA-based PoC devices are yet commercially available, promising research prototypes demonstrate their potential. For example, a paper-based NASBA assay integrated with CRISPR/Cas9 and toehold switch sensors enabled the sensitive detection and differentiation of Zika virus African and American strains from plasma samples with a limit of detection (LOD) of 2.8 femtomolar (fM) [51]. Additionally, the PHANTOM (PHAsed NASBA-Translation Optical Method) system, which combines NASBA with luminescent toehold switch readouts, enables SARS-CoV-2 detection in under 35 min, achieving an LOD of 100 copies per reaction [58]. Another NASBA-toehold switch fluorescence-based sensor has been developed for SARS-CoV-2 detection in nasopharyngeal swabs, demonstrating proof-of-concept programmability for synthetic riboregulatory diagnostics [48].

Compared to LAMP and RPA, NASBA offers high specificity for RNA detection and produces RNA amplicons ideal for transcription-driven biosensing [113]. However, NASBA typically exhibits slower amplification kinetics than LAMP, and its reliance on a multi-enzyme reaction system increases complexity, requiring careful optimization to maintain assay performance under field conditions [114, 115]. Moreover, NASBA is also more susceptible to inhibition from biological matrices, requiring optimized sample preparation and reaction conditions to ensure reliable PoC deployment. Overall, while NASBA holds substantial promise for RNA virus diagnostics at the point of care, further engineering and validation are needed to translate these advanced laboratory prototypes into robust, affordable, and field-deployable PoC platforms suitable for resource-limited environments [58, 116–118].

#### Recombinase polymerase amplification (RPA)

RPA is an isothermal method operating at 37–42 °C, enabling rapid nucleic acid amplification within 20–30 min using minimal instrumentation. The RPA amplification process utilizes a recombinase protein, single-stranded DNA-binding proteins (SSBs), and DNA polymerase to achieve rapid amplification without the need for thermocyclers (Fig. 3c). The mechanism begins with recombinase-primer complexes facilitating primer invasion into the target dsDNA. SSBs then stabilize the displaced DNA strand, allowing DNA polymerase to extend the primer and synthesize the new strand. Its low-temperature requirement, rapid kinetics, and minimal equipment needs make it well-suited for PoC diagnostics in resource-limited settings [119, 120].

RPA has demonstrated broad applicability in infectious disease diagnostics. It has been used effectively for pathogens such as SARS-CoV-2, African swine fever virus (ASFV), malaria parasites, *Mycobacterium tuberculosis*,* Neisseria gonorrhoeae*, and avian influenza viruses (H1–H16 subtypes). For instance, the CoRapID assay combines RT-RPA with LFA to detect SARS-CoV-2 variants (Alpha to Omicron) from swabs with an LOD of 1 copy/reaction and 88.1% sensitivity in under 30 min, representing a true PoC commercial-ready platform [44]. Additionally, the iSCAN-V2 platform integrates RT-RPA with CRISPR/EsCas13d for SARS-CoV-2 detection from swabs, achieving 93.75% sensitivity with a LOD of 8 copies/µL in less than 60 min, making it a PoC-compatible prototype [52]. Furthermore, the smartphone-readable RPA-LFA system was developed for detecting *Leishmania* in blood samples with a sensitivity of 98.7% and a LOD of 0.01 parasites per reaction, making it suitable for PoC applications [30].

Integration with LFA, dipstick formats, and smartphone apps has expanded the utility of RPA in decentralized settings. CRISPR-based detection using Cas12a or Cas13a further enhances specificity for targets such as SARS-CoV-2, HIV, RSV, and rabies viruses [61–63]. A Microfluidic-Integrated RPA system (MI-IF-RPA), which enhances detection efficiency by combining reverse transcription RPA (RT-RPA) with microfluidic lateral flow technology. This method detects SARS-CoV-2 from different swabs with a LOD of 30 copies per reaction and 97% sensitivity in just 30 min. The integration of microfluidics improves portability and reduces reaction time, demonstrating the potential of RPA-driven approaches to revolutionize PoC diagnostics and enable prompt infectious disease management [54]. However, no fully commercialized integrated RPA PoC device has been widely adopted yet.

Despite these promising advancements, commercialization barriers remain, including reagent stability, cold chain requirements, and high enzyme production costs, which limit widespread deployment in decentralized settings. Furthermore, RPA reactions can be inhibited by complex sample matrices such as blood or stool, necessitating robust extraction protocols or inhibitor-tolerant buffer systems to ensure reliable performance in field applications [119, 121, 122].

#### Loop-mediated isothermal amplification (LAMP)

LAMP is a well-established molecular method for amplifying nucleic acids at a constant temperature, typically 60–70 °C, thereby eliminating the need for thermocyclers, similar to NASBA and RPA [123]. The method utilizes a set of 4–6 specific and carefully designed primers that target 6–8 regions of nucleic acid sequence, resulting in loop structures that allow for rapid and effective amplification (Fig. 3d) [123]. This amplification process generates substantial quantities of targeted DNA in 30 to 60 min, which can be detected using turbidity, fluorescence, or colorimetric changes. The high sensitivity, specificity, and isothermal nature of LAMP make it particularly suitable for PoC diagnostics where laboratory infrastructure is limited [29, 124, 125].

Due to its rapid amplification capabilities, LAMP has been extensively applied in the diagnosis of bacterial and viral pathogens, such as *Giardia duodenalis*,* Mycoplasma synoviae*, infectious bronchitis virus (IBDV), Zika virus, and SARS-CoV-2 [126–131]. Its integration with diverse detection platforms further enhances its utility in resource-limited settings by producing rapid and reliable outputs [55]. For instance, Sadeghi et al. (2014) developed a method that integrates an RT-LAMP with the LFA method to diagnose COVID-19 from swabs. This method produces the results within 75–90 min with an LOD of 4 to 40 × 10^-6^ ng/µl [64]. Additionally, the Lucira Check-It Test combines RT-LAMP with colorimetric detection for SARS-CoV-2 and Influenza virus A/B, offering results within 30 min with 93% sensitivity. This test is commercially available as a true PoC device [53]. Furthermore, the RT-LAMP/Toehold switch-based detection system has been developed for detecting MERS-CoV and SARS-CoV-2 in different respiratory tract samples, with an LOD of 120 copies per reaction and results within 70 min [65].

Other LAMP-based PoC systems have been developed or commercialized. For example, the COVIDISC platform integrates RT-LAMP with intercalating dyes (SYTO-82) or fluorescent probes to detect SARS-CoV-2 from nasopharyngeal swabs with an LOD of 1 copy/µL within 20–60 min [45]. The Genie III device offers portable LAMP-based detection for various targets, including nasopharyngeal swabs and serum samples, within 30–60 min, and is widely used in near-PoC settings [49]. Furthermore, innovative detection platforms have been prototyped, such as the pipette-actuated capillary array comb (PAAC) system, which employs fluorescence-based LAMP detection for *E. coli*,* K. pneumoniae*, and *S. aureus* in urine samples, achieving LODs of 200, 500, and 500 copies, respectively, within 85 min [59]. A paper-based microfluidic LAMP system integrated with LFA has been developed for detecting *P. vivax*,* P. malariae*,* and P. ovale* in blood samples, achieving sensitivities of 98% for *Plasmodium* species and 93% for *P. falciparum* within 50 min [57]. These features collectively highlight LAMP’s exceptional suitability for PoC diagnostics. However, challenges such as non-specific amplification necessitate rigorous primer design and optimization to ensure diagnostic accuracy in both clinical and field settings [49, 128, 129, 132, 133].

### Detection modalities in nucleic acid PoC platforms

The continuous advancement of nucleic acid-based diagnostics in PoC testing has allowed for the development of several detection methods that enhance the sensitivity, specificity, and scalability of diagnosing infectious diseases. These detection methods include colorimetric detection, fluorescence-based detection, LFAs, microfluidic and device-based systems. Each detection method provides unique advantages depending on its context of operation and integration with nucleic acid amplification methods such as RPA and LAMP, which enhance the efficiency of PoC testing for a wide range of pathogens, including SARS-CoV-2, HIV, and HPV.

#### Colorimetric detection

Colorimetric detection is widely used in nucleic acid-based PoC diagnostics due to its simplicity and rapid visual readout. This method typically involves a pH-sensitive indicator, a color change associated with a metal nanoparticle or an enzyme-substrate, or an associated color change based on target amplification. For example, a combination of LAMP and a colorimetric detection for MERS-CoV and SARS-CoV-2 viruses represents a low-cost and accessible option for PoC testing [65]. In one such approach, chlorophenol red-D-galactopyranoside (CPRG) undergoes beta-galactosidase catalysis to convert to chlorophenol red (CPR), resulting in a distinct color change [134]. The paper-based cell-free toehold switch biosensor, which utilizes Q5 DNA polymerase and a bioluminescence detection-based toehold switch sensor, also provides a colorimetric readout for determining SARS-CoV-2 in saliva, yielding results within 7 min [56]. Furthermore, the paper-based microfluidics method for DNA diagnostics integrates LAMP with colorimetric detection to diagnose *Plasmodium* species (including *P. vivax*,* P. malariae*,* and P. ovale*), with sensitivities of 98% for *Plasmodium pan* and 93% for *P. falciparum* in blood samples, providing results in under 50 min [57]. Despite its operational simplicity and compatibility with REASSURED criteria, this method faces challenges, including subjective interpretation due to variations in color intensity, lower sensitivity compared to fluorescence-based methods, and potential environmental interferences that may affect assay accuracy [135].

#### Fluorescence-based detection

Fluorescence-based detection provides high sensitivity and specificity, making it a gold standard in molecular diagnostics. This method utilizes fluorescent probes or dyes, such as SYBR Green, for the real-time detection of amplified nucleic acids. Fluorescence detection is widely utilized in amplification platforms such as RT-PCR, RPA, and LAMP [136–138]. For example, COVIDISC is a commercially available PoC diagnostic test, which detects SARS-CoV-2 from nasopharyngeal swabs. This test integrates RT-LAMP with Intercalating dyes (SYTO-82) or fluorescent probes, allowing for the detection of viral infection in under 60 min [45]. Additionally, the RPA-CRISPR/Cas12a-based method for detecting *M. pneumoniae* in sputum employs fluorescence detection, achieving 99.1% sensitivity and an LOD of 2 copies/reaction, with results available within 60 min [62]. Another example is the RPA-CRISPR/Cas12a method for detecting multiple respiratory viruses, such as HuNoV, HmPV, HBoV, SEoV, and RSV, which utilizes a fluorescent readout to ensure high specificity and sensitivity within 40 min [63]. While fluorescence-based detection provides robust and accurate results with potential for automation, its requirement for specialized instrumentation limits accessibility in low-resource settings [45].

#### Lateral flow assay (LFA)

Strip assays, especially LFAs, are commonly used for rapid and equipment-free detection of nucleic acids in PoC diagnostics. This method uses immobilized recognition elements that bind to target nucleic acids, such as gold nanoparticles (AuNPs) or latex beads. This binding produces visible bands on a nitrocellulose membrane [30]. An example is CoRapID, a commercial test that integrates RT-RPA with LFA for SARS-CoV-2 detection, including multiple variants, with an LOD of 1 copy per reaction and 88.1% sensitivity within 30 min [44]. Another example is the RT-LAMP-LFA method for SARS-CoV-2 detection from nasopharyngeal and oropharyngeal swabs, offering 100% sensitivity and results within 75–90 min, making it a reliable PoC-compatible prototype [64]. Additionally, the RPA-CRISPR/Cas13a-based method for detecting H5N1 avian influenza subtypes utilizes LFA to provide rapid results with high sensitivity, making it suitable for field applications [61]. The advantages of LFA include low cost, operational simplicity, stability at ambient temperatures, and minimal training requirements, making it highly suitable for decentralized testing. However, challenges such as limited quantitative capability and batch-to-batch variability remain to be addressed to maximize its diagnostic utility [64].

#### Microfluidic and device-integrated detection

Microfluidic systems and device-based detection methods represent significant innovations in lab-on-a-chip technology, offering automated, miniaturized, and efficient diagnostic platforms. These methods enhance the efficiency and accuracy of detection by minimizing the use of reagents and reducing the time from sample to result. For example, Paper-based microfluidic devices have been developed for detecting the DNA of *P. vivax*,* P. malariae*,* and P. ovale* from blood samples, yielding results in under 50 min [57]. Similarly, the BioFire FilmArray utilizes multiplex PCR in a cartridge system to detect more than 30 pathogens, including RSV, influenza virus, and SARS-CoV-2, in just 60 min [42, 104]. Additionally, the QuantuMDx Q-PoC platform, which integrates RT-PCR with device-based detection, enables the rapid detection of SARS-CoV-2, Flu A/B, and RSV from nasopharyngeal swabs in 30 min [60]. The Cepheid GeneXpert system, a widely used PCR-based platform, detects a variety of targets, including SARS-CoV-2, RSV, and influenza virus, from samples like nasopharyngeal swabs, sputum, blood, and urine, within 60 min [43]. These FDA-approved systems exemplify the potential of device-based diagnostics in clinical and PoC settings. Another example is the iSCAN-V2 platform, which integrates RT-RPA with CRISPR/EsCas13d for SARS-CoV-2 detection, offering results in under 60 min and highlighting its PoC compatibility [52]. Despite their high accuracy and multiplexing capabilities, these methods often require complex manufacturing processes and are associated with higher costs, which may limit their widespread implementation in resource-limited regions [57].

## Part III: The innovation frontier: synthetic biology and digital integration

### Emerging technologies in nucleic acid-based PoC testing

#### CRISPR/cas systems as PoC detection technologies

The CRISPR system was initially identified as an adaptive immune defense mechanism in bacteria and archaea. CRISPR-associated (Cas) proteins recognize and cleave the target nucleic acid sequence, either DNA or RNA [33]. Although initially developed as a gene-editing tool, CRISPR technology has been repurposed for nucleic acid detection, offering rapid, specific, flexible, and portable diagnostics suitable for PoC applications, particularly in resource-limited settings [139]. However, direct application of CRISPR/Cas detection systems without pre-extraction or amplification can result in low sensitivity or false-negative outcomes, necessitating their integration with pre-amplification steps to achieve optimal diagnostic accuracy [140].

CRISPR-based biosensing systems can be broadly categorised into platforms such as DETECTR (DNA Endonuclease Targeted CRISPR Trans Reporter), SHERLOCK (Specific High-Sensitivity Enzymatic Reporter Unlocking), and HOLMES (1-Hour Low-cost Multipurpose Highly Efficient System), each utilising unique Cas proteins to detect diverse pathogens [141, 142]. The most commonly used Cas proteins include Cas9, Cas12, and Cas13, each of which enables distinct detection mechanisms. Although the Cas9 protein lacks collateral activity, Cas9-based diagnostics utilize the accurate and sequence-specific DNA-binding ability of the Cas9 protein [143]. In PoC settings, a dCas9 has been developed to maintain target detection while preventing DNA cleavage. This dCas9 protein can be conjugated with fluorescent reporters, enzymes, or other signalling molecules for direct visualization of nucleic acid targets. This method was achieved by linking dCas9 into a fluorescent reporter in fluorescent in situ hybridization (dCas9-FISH) to detect pathogen DNA in clinical samples [140, 141].

On the other hand, Cas12-based diagnostics utilize the enzyme’s collateral cleavage activity on single-stranded DNA (ssDNA) when it binds to its target double-stranded DNA (dsDNA). For instance, the DETECTR platform integrates Cas12a with isothermal amplification methods, such as RPA and LAMP, to detect pathogens like SARS-CoV-2 [139]. Activated Cas12a cleaves non-specific ssDNA reporters, generating detectable fluorescent signals or lateral flow readouts. Moreover, Cas12-based systems can be coupled with electrochemical sensors, where collateral cleavage alters electrical currents, providing quantifiable signals suitable for portable PoC detection [33, 63]. Similarly, the HOLMES system utilises the collateral cleavage activity of Cas12a and Cas12b, integrating pre-amplification steps to enhance sensitivity and avoid false negatives, enabling rapid detection of both DNA and RNA targets in under an hour [140, 141, 144].

Cas13-based diagnostics are tailored explicitly for RNA detection. Upon binding to target RNA, Cas13 exhibits collateral RNase activity, cleaving surrounding RNA molecules. The SHERLOCK platform harnesses this capability by combining Cas13a with isothermal amplification methods, such as RPA or LAMP, to detect viral RNA from pathogens like SARS-CoV-2 and Zika virus with high sensitivity, generating visual or lateral flow strip readouts [139, 145]. Additionally, Cas13-based assays have been adapted for non-invasive applications, such as saliva-based testing for dengue virus, thereby eliminating the need for blood sampling [32, 139, 140, 143].

These CRISPR/Cas-based diagnostics have advanced PoC testing by enabling rapid, accurate, sensitive and portable detection of infectious diseases. For example, the RPA-CRISPR/Cas12a technique identifies *M. pneumoniae* in oropharyngeal swabs with a sensitivity of 99.1% and an LOD of 2 copies/reaction in under an hour. Similarly, the RPA-CRISPR/Cas13a method has been applied to *M. pneumoniae* and HIV, demonstrating the adaptability of CRISPR systems for diagnosing different pathogens. These systems eliminate the need for advanced laboratory infrastructure, enabling decentralized testing with results that are interpretable via lateral flow strips or smartphone-compatible devices, a significant advantage in resource-limited regions for the rapid identification of pathogens such as tuberculosis, influenza, and norovirus [62, 63, 146].

Several of these technologies have been transformed from lab research into real-world clinical use. This transformation has led to advancements in affordable and portable technologies, such as the DETECTR system (developed by Mammoth Biosciences) and SHERLOCK (developed by the Broad Institute and Sherlock Biosciences). They have developed commercial assays for infectious diseases, such as SARS-CoV-2 and malaria, that deliver results in under 30 min [147, 148]. Notably, SHERLOCK’s COVID-19 test received emergency use authorization (EUA) from the FDA during the pandemic [149].

Despite these advancements, many CRISPR-based PoC diagnostics remain in the clinical validation phase. For example, Sherlock Biosciences’ over-the-counter sexually transmitted infection (STI) test is currently undergoing FDA evaluation in the PROMISE trial (*n* = 2,500), reflecting the gap between technological innovation and regulatory approval. Moreover, real-world implementation faces technical and logistical hurdles, such as minimizing off-target effects, ensuring consistent sensitivity across diverse sample types (e.g., blood, saliva), and scaling production persist. Limitations, such as reagent thermostability, further constrain deployment in low-resource settings where cold chain maintenance is impractical [143, 148].

#### Synthetic RNA sensors and toehold switches

**Fig. 4:**
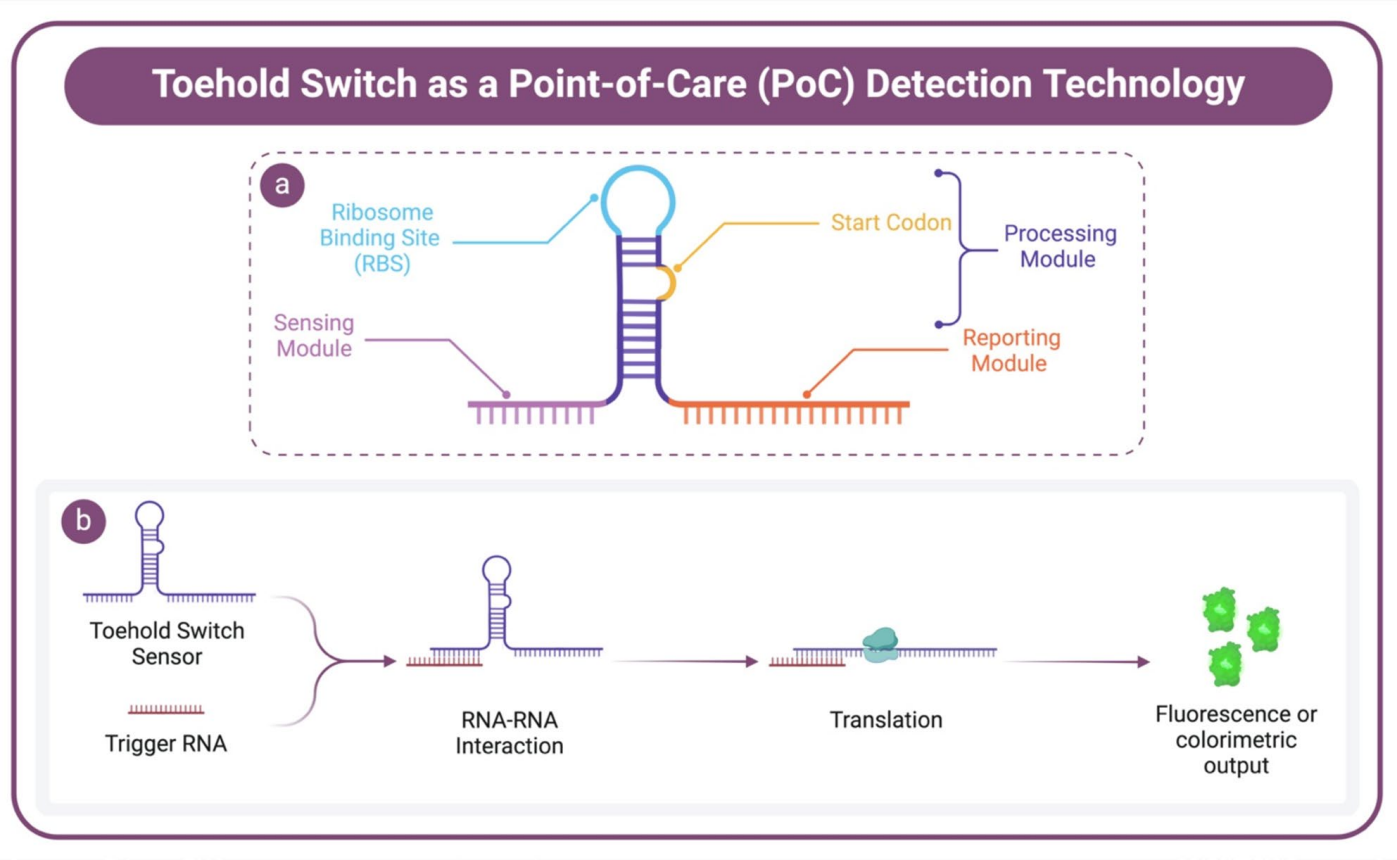
Graphical illustration showing the use of the toehold switch as a point-of-care (PoC) detection technology. **(a)** shows the structural components of the toehold switch, including the sensing module, ribosome binding site (RBS), start codon, processing module, and reporting module. **(b)** represents the detection mechanism using a toehold switch sensor. The toehold switch sensor interacts with trigger RNA, initiating RNA-RNA interaction. This interaction led to translation, resulting in either a fluorescence or colorimetric output, which provided a visual indication of the target’s presence. Created with Biorender.com

The field of molecular diagnostics has recently focused on employing RNA-based regulatory systems, especially riboswitches and toehold switches, as innovative detection methods for infectious diseases. These RNA-based diagnostics offer high sensitivity and specificity, making them attractive candidates for PoC applications [150]. Riboswitches are cis-regulatory mRNA-based elements embedded within mRNA molecules that interact with specific ligands to regulate gene expression by inducing structural alterations, thereby influencing transcription or translation processes [151]. Due to their adaptability, riboswitches have been utilised in various applications, including biosensing, metabolic pathway regulation, and molecular reporting [152, 153]. Toehold switches, in particular, represent a class of synthetic RNA molecules designed with a unique secondary structure comprising three modules (Fig. 4). The sensing module, also known as the toehold region, is typically 15–30 nucleotides in length and serves as a binding site complementary to the target RNA sequence. The processing module comprises a hairpin loop that contains the ribosome binding site (RBS) and the start codon. This module is linked to the reporting module through a specific linker. The reporting modules contain the repressed reporter gene. In the absence of the target RNA, the hairpin structure sequesters the RBS, preventing translation of the reporter gene. Upon binding of the target RNA to the toehold region, the hairpin loop undergoes unwinding, linearising the toehold switch structure. This conformational change exposes the RBS, enabling translation initiation and subsequent expression of the reporter gene, which produces a detectable signal such as fluorescence or a colorimetric change. This programmable and modular design enables precise control of gene expression in response to specific RNA sequences, making toehold switches highly adaptable for diverse diagnostic applications. Their synthetic nature allows for rapid reprogramming against emerging pathogens, supporting their integration into PoC systems for field-deployable, low-cost, and robust infectious disease diagnostics [154, 155].

Significant advancements have been achieved in addressing challenges related to toehold switch design, particularly in terms of increasing efficiency and reducing background activity [156]. Researchers have developed novel approaches using both natural and synthetic riboswitches to engineer the aptamer and expression platforms, thereby expanding their adaptability and utility across diverse applications [157, 158]. The design of toehold switches depends heavily on computational tools to predict RNA folding and optimize sequence specificity. In silico modeling enables researchers to simulate the secondary structure of toehold switches and their interactions with target pathogenic RNA sequences, thereby ensuring minimal off-target effects and high binding efficiency [159]. Tools such as NUPACK and ViennaRNA, along with other recently developed design software summarised in (Table 3), are commonly employed to design toehold switches with optimal performance. Furthermore, computational design facilitates the creation of multiplexed systems capable of detecting multiple pathogens simultaneously, further enhancing their diagnostic effectiveness [160, 161].

**Table 3:** List of recently released and well-established riboswitch and toehold switch design tools

Tool	Description	Availability	References
RiboLogic	Automated design of riboswitches optimizing sequence and structure	Open-source	[162]
MODENA	Genetic algorithm-based ON riboswitch design	web server	[163]
Toeholder	Automated design and validation of toehold switches	Open-source	[164]
NUPACK	Nucleic acid structure analysis and design suite	Open-source + web server	[160]
ViennaRNA Package	RNA secondary structure prediction and analysis package	Open-source	[161]

One of the most remarkable characteristics of toehold switches is their programmability. These synthetic RNA sensors can be easily reconfigured to target different RNA sequences by altering only the sequence of the sensing module [165–169]. Their ability to integrate with different readout systems, including fluorescence, colorimetric assays, and electrochemical sensors, broadens their applicability across diagnostic platforms. Importantly, integration with cell-free expression systems and paper-based platforms makes toehold switches particularly suitable for resource-limited settings [49, 170, 171]. Due to its versatility, toehold switch technology is considered an innovative technology for PoC diagnostics for infectious diseases. They provide a rapid, accurate, and sensitive detection platform capable of identifying pathogenic RNA with high specificity, especially when combined with isothermal amplification methods such as LAMP, RPA, and NASBA. For example, scientists developed a method during the COVID-19 pandemic that integrates a paper-based toehold switch method with RT-LAMP to identify SARS-CoV-2 in just 70 min [65]. Toehold switches have also been successfully used to identify various viruses such as the Zika virus, the Ebola virus, and the respiratory syncytial virus subgroups A and B, highlighting their broad applicability and sensitivity for managing infectious disease outbreaks [172]. Beyond viral detection, toehold switches have been used to detect bacterial pathogens, including *M.* ***tuberculosis***, which causes tuberculosis, by targeting specific microRNA sequences [173].

Furthermore, these RNA-based biosensors have been utilized to identify genes associated with antibiotic resistance, which are crucial for guiding effective antimicrobial therapy [168]. One example is a toehold switch-based system designed to detect and analyze gut microbiota and host biomarkers, with potential applications in both pathogen detection and microbiome profiling [174]. Moreover, toehold switches have also been validated for cancer diagnosis by targeting oncogenic RNA signatures in patient samples, displaying their potential for broader application beyond infectious diseases. Due to their affordability, portability, and rapid response time, toehold switches align with the WHO REASSURED criteria, which speaks highly of their significant potential in decentralized diagnostic applications [167, 169, 175].

Additional features in the toehold switch-based detection assay, such as freeze-dried and shelf-stable formulations, have significantly increased their usefulness in resource-limited settings. For example, a freeze-dried toehold switch assay for Zika virus detection remained stable and reliable even under tropical conditions in high-temperature hotspots [171, 176]. With continued research and development, toehold switches hold the potential to deliver a platform foundation for next-generation molecular diagnostics, advancing decentralized and accessible healthcare solutions globally. The increasing interest in toehold switches has also led to substantial intellectual property developments. Numerous patents have been filed covering their design, optimization, and application in PoC diagnostics (Table 4). For example, scientists at Harvard University’s Wyss Institute hold key patents about the application of toehold switches in detecting viral RNA, reflecting their market potential. Companies are actively exploring the integration of their products into portable diagnostic devices, and the development of freeze-dried, shelf-stable formulations has further facilitated their commercialization for global health initiatives [177–179].

**Table 4:** Patents on riboswitches and toehold switches for point-of-care (PoC) diagnostics

Patent Title	Patent Number	Description	Assignee/Inventor(s)	References
Unimolecular aptamer-based sensors for pathogen detection	US20240052400A1	RNA aptamer-based sensors for detecting pathogen markers with a simple output signal	Arizona State University	[180]
Portable, low-cost pathogen detection and strain identification platform	US12116623B2	A portable diagnostic platform using synthetic RNA switches for rapid pathogen detection	Massachusetts Institute of Technology Harvard University Arizona State University	[177]
Loop-Mediated Synthetic Riboregulators	US20240018608A1	Synthetic RNA devices using loop-mediated regulation for pathogen RNA detection	Arizona State University	[181]
Compositions comprising riboregulators and methods of use thereof	US11788156B2	RNA riboregulators (toehold switches) that detect nucleic acid triggers for diagnostics	Boston University Harvard University	[182]
Ultraspecific riboregulators having robust single-nucleotide specificity and in vitro and in vivo uses thereof	US20240043853A1	High-fidelity RNA riboregulators for detecting single-nucleotide variants in pathogens	Arizona State University	[183]
Synthetic translation-sensing riboswitches and uses thereof	US10550440B2	RNA-based switches that regulate translation in response to specific target sequences	Arizona State University	[184]
Integrated diagnostic devices having embedded biomolecular computing systems and uses thereof.	US11547997B2	Devices incorporating synthetic biology-based logic for automated pathogen detection	Arizona State University	[178]
Ultraspecific Nucleic Acid Sensors for Low-Cost Liquid Biopsies	US20220372586A1	RNA-based sensors for detecting trace nucleic acids in liquid biopsies for diagnostics	Arizona State University	[179]
Rapid low-cost detection of valley fever using isothermal amplification and sensing methods	US11214841B2	Isothermal amplification combined with RNA sensors for valley fever diagnostics	Arizona State University	[185]

Despite their potential, toehold switches face various challenges. One primary concern is their sensitivity to low-abundance RNA targets, especially during early-stage infections. Furthermore, the design and optimization process of toehold switches can be time-consuming and require significant computational resources. Off-target interactions and background activity can also affect their performance, necessitating thorough validation. Future studies aim to address these limitations by advancing computational modeling approaches, improving signal amplification techniques, and integrating toehold switches with CRISPR-based systems to achieve greater specificity [56, 65].

### Proposed innovative technologies for nucleic acid-based PoC diagnostics

The continuous growth of emerging pathogens, rising antimicrobial resistance, and pandemic threats such as COVID-19 underscore the urgent need for the development of PoC diagnostics that are rapid, accurate, cost-effective, and accessible, particularly in resource-limited settings [1]. Although there is a large number of PoC diagnostics, only a few of them meet the WHO’s REASSURED criteria, indicating the need for ongoing development to achieve levels of accuracy comparable to traditional laboratory-based methods such as PCR [4]. Therefore, we consider laboratory PCR as the reference standard for analytical performance, but we also stress that emerging PoC systems are designed to complement centralized laboratory testing by extending diagnostic capacity into resource-limited settings. This gap has driven innovation in synthetic biology, which integrates systems such as toehold switches and CRISPR/Cas systems with various isothermal amplification methods to develop next-generation systems. These integrated systems promise to achieve laboratory-grade accuracy in portable and straightforward formats [33, 65, 170], enabling the simultaneous detection of multiple pathogens, strain differentiation, and rapid deployment during outbreaks or pandemics. For example, Pardee et al. (2016) demonstrated a two-stage diagnostic platform for the Zika virus that integrated NASBA as an isothermal amplification method with toehold switch sensors and a CRISPR/Cas9 detection system. This two-stage system enables simultaneous wide pathogen screening and high-resolution strain differentiation. NASBA amplified viral RNA at a constant temperature, and the amplified product was subsequently detected using freeze-dried, paper-based toehold switches that produced a visible colorimetric change (from yellow to purple) upon binding to target sequences. After achieving the colorimetric changes, the amplified product was added to the CRISPR/Cas9 system to differentiate between African and American Zika virus strains by targeting strain-specific mutations. In addition to differentiating Zika strains, this system aligns with WHO’s REASSURED criteria, costing less than $1 per test, requiring no cold storage, and delivering results within 90 min. These innovations demonstrate the power of synthetic biology for PoC diagnostics, though integrating all components into a single portable device remains challenging [51].

**Fig. 5:**
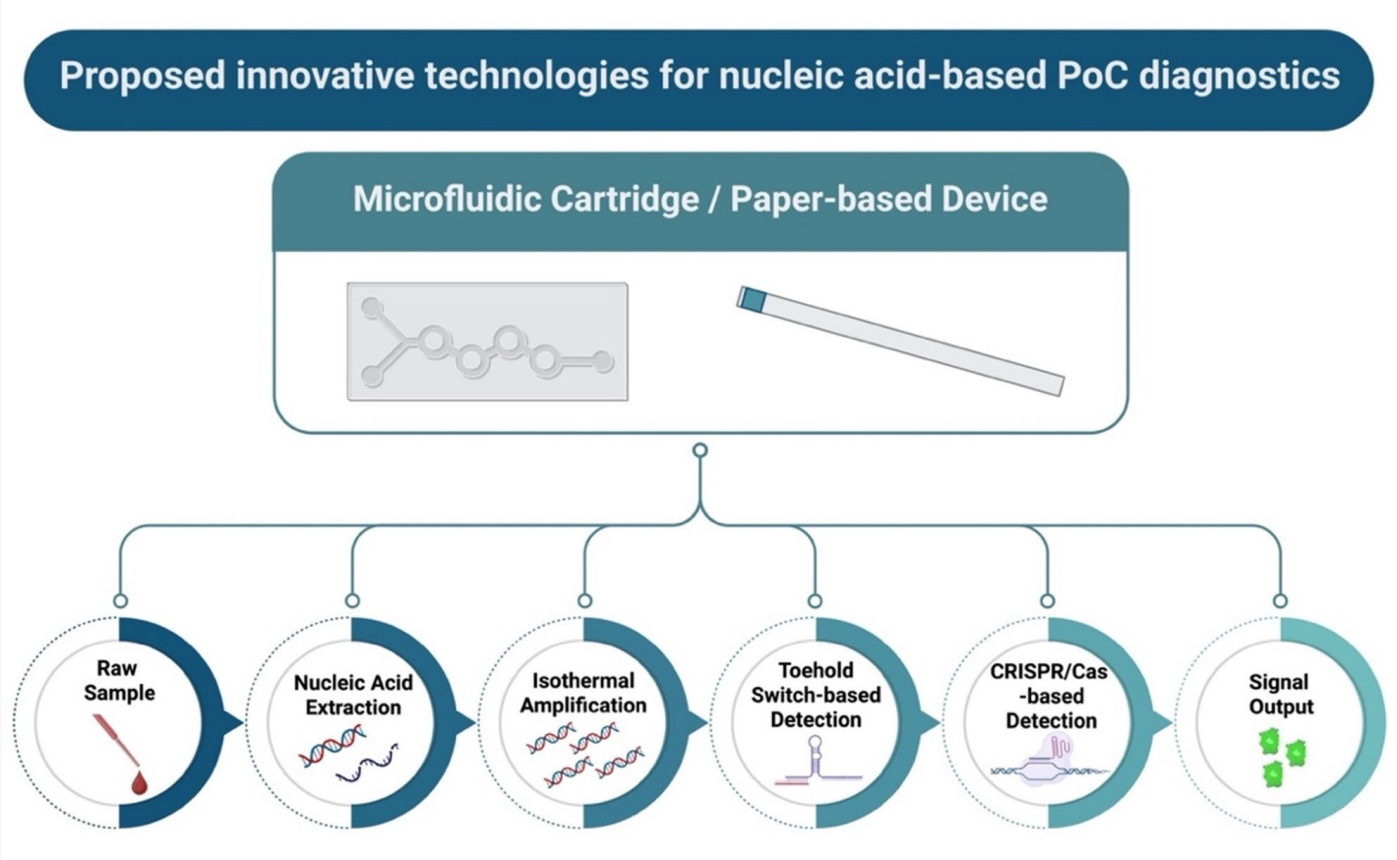
Proposed innovative technologies for nucleic acid-based point-of-care (PoC) diagnostics**.** created with Biorender.com

A proposed diagnostic platform combines the accuracy of a microfluidic system with the simplicity of paper-based systems to create a lab-on-a-chip diagnostic system (Fig. 5). This lab-on-a-chip system facilitates the detection process by automating all steps, from sample preparation to result interpretation, in a compact and portable device. At its core, the system integrates four main modules: nucleic acid extraction, isothermal amplification, and CRISPR or toehold-based detection. For the nucleic acid extraction, Silica membrane-based microfluidic channels offer a cost-effective and user-friendly approach for nucleic acid extraction to be employed in PoC diagnostics. This method has been validated for HBV and HIV RNA isolation, demonstrating comparable performance to commercial instrument extraction, achieving RNA isolation in under one minute from serum samples. By contrast, traditional column-based methods are less suited to PoC applications due to higher costs and operational complexity [77]. The isothermal amplification method is the next essential step in the system. NASBA and RPA are the most suitable methods for this system as they operate at a constant temperature.

Additionally, NASBA is particularly advantageous for RNA viruses (e.g., HIV, SARS-CoV-2), as it directly amplifies RNA, thereby bypassing the reverse transcription steps required in PCR [31, 186]. RPA is better suited for DNA targets and offers rapid results [30, 62]. Both methods eliminate the need for thermocyclers, enabling compatibility with portable and battery-powered heaters. Recent advances in lyophilized reagents further enhance their suitability for PoC use, as pre-loaded amplification mixes remain stable for months at different temperatures [110]. The detection and strain differentiation step can be employed using a two-step approach: toehold switch sensors for initial screening and CRISPR-Cas systems for confirmation, as proposed by Pardee et al. (2016) [51]. Toehold switches produce a visible color change when they detect target RNA, making them a low-cost and easy-to-use option. For higher specificity, CRISPR/Cas12a or Cas13a enzymes cleave reporter molecules only when the target sequence is present, allowing the detection of specific strains [56, 62, 65, 139, 144, 170, 172]. A dual-channel microfluidic design can separate screening and confirmation, improving accuracy. However, integrating these three technologies into a single microfluidic or paper-based device remains challenging, particularly in minimizing preparatory steps and reducing contamination risks. Freeze-dried reagents can be pre-loaded onto paper strips or embedded into cartridges compatible with simple handheld readers or smartphone-based imaging systems [187]. This scalability enables rapid adaptation to emerging pathogens, making the platform highly scalable and versatile for future pandemics or endemic disease surveillance.

### Integration with digital health and artificial intelligence (AI)

The integration of PoC testing with digital health technologies and artificial intelligence (AI) enhances the diagnostic approach to infectious diseases. Additionally, these PoC tests rely on manual interpretation of the results, which involves judging the faint test lines of the LFA, thereby limiting the scalability of PoC diagnostics. Traditional PoC tests typically rely on manual reading of faint LFA test lines, which introduces inter operator variability and constrains scalability, especially when deployed at population level. they also provide rapid and decentralized diagnostic capabilities, but lack the digital connectivity and intelligent data analysis tools that make it smarter and suitable for real-time decision-making. These features enhance the value of PoC diagnostics by increasing their availability in resource-limited settings. Digital health platforms, including mobile health applications and cloud-based systems, have significantly enhanced the efficiency of PoC diagnostics by enabling automated result upload, time–location stamping, and integration with clinical workflows [188]. Christodouleas et al. (2018) describe “eDiagnostics” architectures in which PoC sensors are coupled to smartphones or dedicated readers that digitize signals (optical, electrochemical, or colorimetric) and transmit them via wired or wireless links to centralized servers for further processing. These systems use embedded algorithms to perform basic preprocessing, such as background subtraction, color normalization, and thresholding. Additionally, there are more advanced models (including early supervised classifiers) can run in the cloud to stratify risk or trigger alarms [188]. For example, smartphone-based diagnostics can detect different pathogens, such as SARS-CoV-2, by introducing a smartphone-based AI framework to trace pulmonary inflammation in COVID-19 cases through ferning pattern analysis of air-dried sputum samples using a low-cost, smartphone-compatible microscopy device. In this study, sputum samples are air dried on a slide to form electrolyte dependent “ferning” patterns whose morphology correlates with pulmonary inflammation. smartphone-compatible microscope captures high resolution images that are processed by a convolutional neural network (CNN) trained end to end on labelled CT positive and CT negative cases. The CNN automatically learns hierarchical features (edges, branching angles, crystal density, and global pattern geometry) without manual feature engineering. The network uses supervised learning with cross entropy loss and standard data augmentation (e.g., rotations, flips, brightness changes) to improve robustness to acquisition variability across devices and users. After training the model, the CNN runs locally on the smartphone to output a probability score of inflammation, achieving around 95% diagnostic accuracy relative to CT and effectively replacing the need for tomographic imaging in many triage scenarios. This paper presents the demonstration of an accessible, deep learning-powered mobile platform for diagnosing respiratory inflammation, which does not require CT infrastructure or extensive sample analysis [189].

The rapid growth of the COVID-19 pandemic highlighted the importance of introducing new technologies such as artificial intelligence (AI) and machine learning (ML) into PoC diagnostics to enhance the accuracy, sensitivity, and efficiency of diagnostic sensors. ML is being integrated into different PoC tests, including LFAs, NAATs, and image-based sensors [190]. For example, University College London and the Africa Health Research Institute developed a diagnostic system for HIV, which integrated the LFA with ML models to enhance the efficiency and accuracy of results interpretations. In this system, field workers use tablets to capture standardized images of HIV LFAs, which are then processed by a deep learning classifier trained on a library of 11,374 real-world images collected in rural South Africa. The study benchmarked support vector machines with hand crafted intensity and texture features against several CNNs and selected a MobileNetV2 based model as the best balance between accuracy and efficiency for on device use. The network’s lightweight architecture, combined with supervised training using cross entropy loss, class weighting, and standard augmentation, enabled robust discrimination of test and control lines under variable imaging conditions. It showed an increase in specificity from 89% to 100% and sensitivity from 95.6% to 97.8% compared with human readers. The same models were later applied to over 500,000 COVID-19 tests in the Real-time Assessment of Community Transmission-2 (REACT-2) study [191, 192]. In a notable advancement, Roh et al.. (2020) introduced a CRISPR/Cas12 platform featuring spatially encoded hydrogel microparticles (HMPs) for multiplexed detection. Each microparticle has its own geometric “face code” and is loaded with Cas12a–guide RNA complexes for specific HPV targets. When a target is present, Cas12a cuts fluorogenic reporters and that particle lights up. As these fluorescent particles flow through the microfluidic channel, a Mask R CNN model finds each one in the image, traces its outline, and assigns a type based on its code and brightness. Because Mask R CNN adds a dedicated mask prediction branch to the usual detection pipeline, it can separate even crowded particles cleanly. The system achieved 97.9% accuracy and a 97.8% F1 score, reliably distinguishing four HMP types while maintaining a detection limit of 2 aM [193].

Despite these advancements, several challenges face the widespread use of AI in PoC diagnostics. Achieving high analytical sensitivity and precision, detecting low-abundance biomarkers, and ensuring diagnostic accuracy comparable to conventional laboratory settings remain primary concerns. Additionally, issues related to regulatory approval, data privacy, and the need for large, high-quality datasets for training AI models must be addressed. Overcoming these challenges is crucial for the successful integration of AI and ML into PoC diagnostic systems, ultimately leading to more accessible and reliable diagnostics for infectious diseases [190].

## Conclusion

PoC molecular diagnostic technologies have transformed the future of infectious disease diagnosis by providing rapid, accurate, and accessible testing, particularly in resource-limited settings. However, the choice between protein-based and nucleic acid-based diagnostics remains crucial, especially in pandemic scenarios. Protein-based diagnostics, including LFAs, provide immediate results, are often less costly, and are most appropriate for mass screening; however, they are less sensitive. In contrast, nucleic acid-based diagnostics, including RT-PCR and isothermal amplification techniques, have high specificity and early detection ability, but are more complex to process and have higher operational costs. The integration of nucleic acid-based diagnostics with novel detection technologies, including CRISPR/Cas and toehold switch technologies, has the potential to reduce costs while enhancing sensitivity and specificity. However, regulatory approval becomes a concern, as long validation delays implementation, and a few tests are retracted post-approval due to performance concerns. For example, the Cue Health COVID-19 test got initial authorization but was later withdrawal once its reliability came under review, identifying challenges in balancing speed and accuracy in emergency approvals. Commercialization also faces challenges. Scalable mass production, on its own, faces constraints in manufacturing processes and supplies. Most PoC kits require temperature control, which hinders their distribution in low-resource areas. Environmentally, concerns are associated with the waste generated from single-use PoC devices, highlighting the need for more sustainable solutions. Ultimately, while rapid development is crucial during pandemics, ensuring clinical validation across diverse populations remains essential to guarantee accuracy and maximize public health impact.

## Data Availability

No datasets were generated or analysed during the current study.
